# Supplementary material for: Soil Chemical and Microbiological Properties Are Changed by Long-Term Chemical Fertilizers That Limit Ecosystem Functioning
Source: Microorganisms. 2020 May 8;8(5):694. doi: 10.3390/microorganisms8050694 (PMC7285516; doi:10.3390/microorganisms8050694)
Supplement: Supplementary file 1 [file microorganisms-08-00694-s001.zip › Supplemental Figure-777489.docx]

**Supplemental Material:**

**Supplementary Figure S1** The samples collection approach used for collecting walnut trees bulk soil, rhizosphere and fine roots. The samples were collected from the 2 ordinate directions of canopy (shady and sunny sides of tree) approximately 3 meters away from the trunk.

**
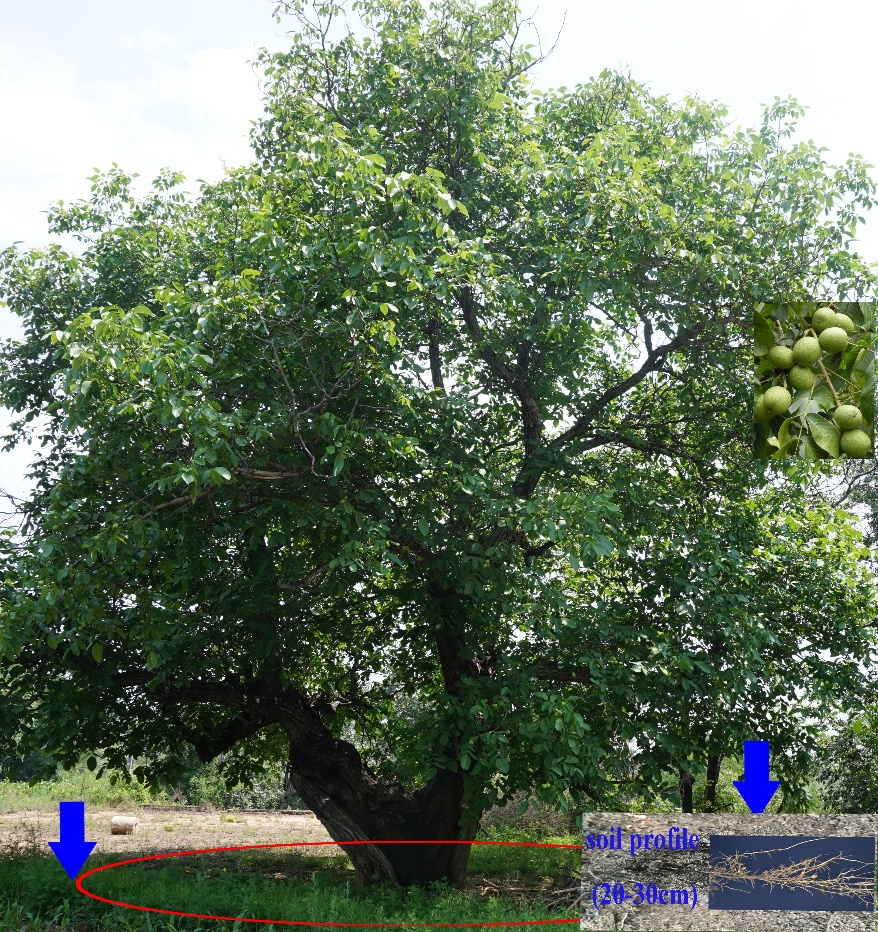
**

**Supplementary Figure S2** Root structure of walnut under stereomicroscope (fleshy and without root hairs).

**
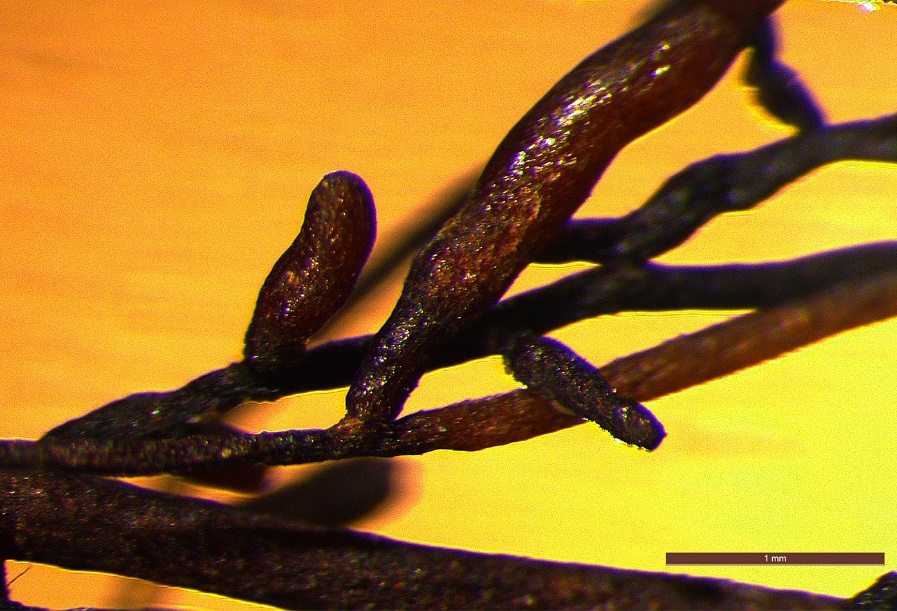
**
